# Revisiting Standard Percutaneous Nephrolithotomy in the Mini-Percutaneous Nephrolithotomy/Retrograde Intrarenal Surgery Era: A Retrospective Audit

**DOI:** 10.7759/cureus.71847

**Published:** 2024-10-19

**Authors:** Nakul Aher, Hariharasudhan Sekar, Natarajan Kumaresan, Sriram Krishnamoorthy

**Affiliations:** 1 Urology, Sri Ramachandra Institute of Higher Education and Research, Chennai, IND

**Keywords:** complications, mini-percutaneous nephrolithotomy (mini-pcnl), modified clavien-dindo scoring system, percutaneous nephrolithotomy (pcnl), risk factors

## Abstract

Objective

This study aims to identify the risk factors associated with postoperative complications following standard percutaneous nephrolithotomy (PCNL) for better results with the emergence of mini-percutaneous nephrolithotomy (mini-PCNL)/retrograde intrarenal surgery (RIRS) in recent years.

Methodology

This retrospective study was conducted in the Department of Urology, Sri Ramachandra Institute of Higher Education and Research, tertiary health care center in Chennai, India, from January 2018 to December 2023. Records of demographic information, along with clinical presentations like any urinary tract infection (UTI), hematuria, or loin pain were recorded. Medical and surgical history noted. Characteristics such as stone burden, location, number of calyces involved, and partial or complete staghorn were noted. Patients had undergone standard PCNL, either prone or supine, as per the preference of the surgeon. Operative time, number of percutaneous puncture tracts, and other intraoperative factors were investigated. A modified Clavien-Dindo scoring system for PCNL was used to classify any complication that occurred during this time. All patients were assessed after one month for stone clearance status.

Results

IBM SPSS Statistics for Windows, version 29 (IBM Corp., Armonk, NY, USA) was used for statistical analysis. The logistic regression analysis results show that age >55 years, urinary tract infection (UTI), diabetes, CKD, stone burden, percutaneous access number >1, and Amplatz sheath size >30Fr are highly significant predictors of complications (p <0.05). A greater number of calyces involved significantly increases the odds of complications. The odds ratio is 2.307 with a significant p-value (0.045), indicating a significant association. Longer operative time substantially increases the odds of complications (odds ratio is 0.129 with p = 0.021).

Conclusion

Standard PCNL has a low overall complication rate and is still an excellent modality of treatment for large-burden kidney stones in the mini-PCNL/RIRS era. Preoperative optimization, reduction in percutaneous access tracts with smaller Amplatz sheath (24Fr), and shorter operative time will help to reduce the complications rate.

## Introduction

Urolithiasis affects roughly 10% of people throughout their lifespan [1]. Additionally, over the past 10 years, it has become increasingly prevalent globally [2]. Percutaneous nephrolithotomy (PCNL) plays a key role in managing large renal calculi but the procedure is not devoid of risks, with postoperative complications becoming significant concerns for both patients and healthcare providers [3].

Chronic kidney function impairment may result from staghorn calculi, highlighting the clinical significance of its management. The conventional therapy for large kidney stones >2 cm in diameter and staghorn calculi is PCNL. A better stone clearance rate is observed with Percutaneous nephrolithotomy compared to extracorporeal shock wave lithotripsy [4]. The reported range of PCNL complication rates is 20-83%. The majority of PCNL consequences are minor, but serious complications like hemorrhage, infection, pleural space violation, and damage to neighboring organs have also been documented [5]. Over the years, researchers have studied many aspects of PCNL and its associated complications, contributing to the knowledge in this domain [6].

In recent years with the emergence of mini-percutaneous nephrolithotomy (mini-PCNL), a less invasive variant, shifts in clinical practices have been observed aiming at reducing these complications and improving patient outcomes. Current literature that audits the complications of conventional PCNL in this era of mini-PCNL often lacks the depth of multivariate analyses. Understanding the risk factors associated with postoperative complications following PCNL is important for better results.

Surgery-related problems are frequently graded using the Clavien-Dindo system. Recent research has confirmed and expanded the usage of a modified Clavien-Dindo classification system to identify PCNL-specific complications [7,8]. We provide the outcomes of PCNL at our tertiary care center, the Department of Urology, Sri Ramachandra Institute of Higher Education and Research, Chennai, aiming at the identification of risk factors for postoperative complications. These determinants encompass a broad spectrum of factors, ranging from patient demographics to stone characteristics, anatomical considerations, surgical techniques, and perioperative management strategies [9]. By analyzing data from conventional PCNL at our institute, we seek to evaluate the validity of transitioning towards mini-PCNL based on empirical evidence and offer clinicians a holistic overview of postoperative complications in PCNL. This study does not compare the safety and efficacy of standard PCNL with mini-PCNL/retrograde intrarenal surgery (RIRS).

## Materials and methods

We conducted this study at the Department of Urology, Sri Ramachandra Institute of Higher Education and Research, Chennai, a tertiary care referral teaching institution in South India.

All patients who underwent standard PCNL from January 2018 to December 2023 were included in this research. The exclusion criteria were as follows: (1) patients with improper documentation, lack of medical records, and no details of surgical procedures were excluded from the study; (2) all patients who had undergone mini-PCNL were excluded as the main focus of this study was to assess the efficacy and safety of standard PCNL; (3) surgeries performed by urologists with less than five years of experience were excluded from the study.

PCNL surgeries performed by three consultant urologists with at least five years of experience in both supine and prone PCNLs were evaluated. Before the surgery, each patient had undergone an evaluation, and records of their demographic information, along with clinical presentation like any urinary tract infection (UTI), hematuria, or loin pain were recorded. All patient's medical and surgical history was noted.

Patients had undergone standard PCNL, either prone or supine, as per the preference of the surgeon. All of the patients underwent routine hematological and biochemical investigations along with non-contrast computed tomography (CT) of the kidney, ureters, and bladder (KUB). Stone characteristics such as stone burden, location, number of calyces involved, and partial or complete staghorn were noted. The stone burden was calculated by the Ellipsoid’s formula (stone volume = 0.167 × π × Length × Width × Depth) as per European Association of Urology (EAU) Recommendations [10]. Coronal and sagittal reconstructed views of non-contrast computed tomography (CT) of the kidney, ureters, and bladder (KUB) were mainly used to assess the dimension of the stones, depending on which stone burden was estimated. The stones were arbitrarily categorized for the study's purposes based on the stone burden, as small (<5000 mm3), intermediate (5000 mm3-20000 mm3 with single calyx involvement), large (5000 mm3-20000 mm3 with >1 calyx involvement), very large (>20000 mm3) stones.

All patients had undergone PCNL after having a ureteral catheter inserted for retrograde pyelogram (RGP). Under fluoroscopic guidance, puncture was performed by a bull's-eye or triangulation technique. A range of PCNL tract diameter with Amplatz sheath size was 24 to 32 Fr. We have used the EMS Swiss LithoClast 2 (EMS Electro Medical Systems SA, Nyon, Switzerland) with a 3.2 mm probe for pneumatic lithotripsy. Operative time, number of percutaneous puncture tracts, and other intraoperative factors were investigated. Following surgery, kidney profiles and blood counts were acquired in clinically indicated patients. Sufferers were treated with intravenous fluids, antibacterial agents, and analgesics following surgery. Once they became medically fit, they were discharged. A modified Clavien-Dindo scoring system for PCNL was used to classify any complication that occurred during this time. Complications were managed and treated safely. Major problems were those, which were categorized as Clavien-Dindo classification of 3a, 3b, 4, or 5.

All patients were assessed after one month. The lack of calculi or the presence of clinically insignificant residual fragmentation (CIRF) on a non-contrast CT scan were considered signs of effectiveness. Calculi that are less than 4 mm in diameter, non-obstructive, asymptomatic, and contagious are referred to as CIRFs.

Among patients who met every requirement for participation, a final evaluation was performed. The collected data were entered in Microsoft Excel 2016 (Microsoft Corp., Redmond, WA, USA) and analyzed with IBM SPSS Statistics for Windows, version 29 ( (IBM Corp., Armonk, NY, USA). To describe the data descriptive statistics frequency analysis, percentage analysis was used for categorical variables and the mean and SD were used for continuous variables. To find the significant difference between the bivariate samples in independent groups the independent sample t-test was used. To find the significance in qualitative categorical data, the Chi-square test was used; similarly, if the expected cell frequency was less than 5 in 2×2 tables, then Fisher's exact was used. To find the predictors of the complications, multivariate analysis by binary logistic regression with the Backward Wald Method was used. In all the above statistical tools the probability value of 0.05 is considered a significant level.

## Results

During the study period, a total of 1760 patients underwent PCNL, of which 229 were excluded due to missing data and lack of proper medical records. The remaining 1531 standard PCNL surgeries, which were performed only by consultant urologists with at least five years of post-training experience in both supine and prone PCNLs, were assessed.

The descriptive data of the patients comparing the complications in the quantitative variable is summarized in Table 1. Patients with complications were older (58.6 ± 12.5 years), and had a higher body mass index (BMI) (25 ± 3.2), a greater stone burden, longer operative times, and longer hospital stays. All these differences were statistically significant with p-values well below the 0.01 threshold, indicating highly significant results.

**Table 1:** Univariate analysis and comparison of postoperative complications of quantitative variable. ** Highly significant at p < 0.01.

Variable	Postoperative complications			
	Present	Absent	t-value	p-value
Age (years)	58.6 ± 12.5	52.1 ± 8.7	7.844	0.0005 **
BMI (body mass index)	25 ± 3.2	23.5 ± 3.2	6.913	0.0005 **
Stone burden (mm^3^)	15813.8 ± 12024	4340.5 ± 1291.6	15.160	0.0005 **
Operative time (In minutes)	99.3 ± 22.6	58.5 ± 13.1	27.926	0.0005 **
Length of hospital stay (days)	3.7 ± 1.9	2.3 ± 0.5	12.032	0.0005 **

The overall complication rate was 16.5%. Table 2 lists a comparison of postoperative complications in the qualitative variable. The presence of complications was significantly higher in males {199 (78.7%)} compared to females {54 (21.3%)} with a p-value of 0.0005. Flank pain was more common in patients with complications {204 (80.6%)} compared to those without {946 (74%)}, with a p-value of 0.026. Hematuria was observed in 102 (40.3%) patients with complications versus 356 (27.9%) without, showing a significant difference (p = 0.0005). Urinary tract infections (UTIs) were significantly more frequent in patients with complications {151 (59.7%)} compared to those without {124 (9.7%)} with a p-value of 0.0005. Hypertension {132 (52.2%) vs. 148 (11.6%)}, diabetes {201 (79.4%) vs. 89 (7%)}, ischemic heart disease {53 (20.9%) vs. 1 (0.1%)}, and chronic kidney disease {66 (26.1%) vs. 44 (3.4%)} were all significantly more prevalent in patients with complications, each with a p-value of 0.0005.

**Table 2:** Univariate analysis and comparison of postoperative complications of qualitative variable. PCNL: percutaneous nephrolithotomy; URSL: ureteroscopic lithotripsy; ASA: American Society of Anesthesiologists. * Significant at p < 0.05 but >0.01; ** Highly significant at p < 0.01; # No statistical significance at p > 0.05.

Variable		Postoperative complications			
		Present (N (%))	Absent (N (%))	Total (N (%))	p-value
Gender	Female	54 (21.3)	471 (36.9)	525 (34.3)	0.0005 **
	Male	199 (78.7)	807 (63.1)	1006 (65.7)	
Flank pain	Yes	204 (80.6)	946 (74)	1150 (75.1)	0.026 *
	No	49 (19.4)	332 (26)	381 (24.9)	
Hematuria	Yes	102 (40.3)	356 (27.9)	458 (29.9)	0.0005 **
	No	151 (59.7)	922 (72.1)	1073 (70.1)	
Urinary tract infection	Yes	151 (59.7)	124 (9.7)	275 (18)	0.0005 **
	No	102 (40.3)	1154 (90.3)	1256 (82)	
Hypertension	Yes	132 (52.2)	148 (11.6)	280 (18.3)	0.0005 **
	No	121 (47.8)	1130 (88.4)	1251 (81.7)	
Diabetes	Yes	201 (79.4)	89 (7)	290 (18.9)	0.0005 **
	No	52 (20.6)	1189 (93)	1241 (81.1)	
Ischaemic heart disease	Yes	53 (20.9)	1 (0.1)	54 (3.5)	0.0005 **
	No	200 (79.1)	1277 (99.9)	1477 (96.5)	
Chronic kidney disease	Yes	66 (26.1)	44 (3.4)	110 (7.2)	0.0005 **
	No	187 (73.9)	1234 (96.6)	1421 (92.8)	
History of shockwave lithotripsy	Yes	18 (7.1)	94 (7.4)	112 (7.3)	0.893 #
	No	235 (92.9)	1184 (92.6)	1419 (92.7)	
History of PCNL	Yes	25 (9.9)	60 (4.7)	85 (5.6)	0.001 **
	No	228 (90.1)	1218 (95.3)	1446 (94.4)	
History of URSL	Yes	59 (23.3)	89 (7)	148 (9.7)	0.0005 **
	No	194 (76.7)	1189 (93)	1383 (90.3)	
ASA class	I	59 (23.3)	971 (76)	1030 (67.3)	0.0005 **
	II	122 (48.2)	307 (24)	429 (28)	
	III	72 (28.5)	0 (0)	72 (4.7)	
Number of calyces involved	Nil	5 (2)	466 (36.5)	471 (30.8)	0.0005 **
	1	116 (45.8)	700 (54.8)	816 (53.3)	
	2	70 (27.7)	44 (3.4)	114 (7.4)	
	3	62 (24.5)	68 (5.3)	130 (8.5)	
Stone burden	Small	33 (13)	1030 (80.6)	1063 (69.4)	0.0005 **
	Intermediate	89 (35.2)	157 (12.3)	246 (16.1)	
	Large	79 (31.2)	91 (7.1)	170 (11.1)	
	Very large	52 (20.6)	0 (0)	52 (3.4)	
Staghorn calculus	Yes	118 (46.6)	121 (9.5)	239 (15.6)	0.0005 **
	No	135 (53.4)	1157 (90.5)	1292 (84.4)	
Percutaneous access number	1	109 (43.1)	1173 (91.8)	1282 (83.7)	0.0005 **
	> 1	144 (56.9)	105 (8.2)	249 (16.3)	
Prone/supine PCNL	Prone	208 (82.2)	1006 (78.7)	1214 (79.3)	0.210 #
	Supine	45 (17.8)	272 (21.3)	317 (20.7)	
Puncture site (lower calyx)	Yes	205 (81)	1213 (94.9)	1418 (92.6)	0.0005 **
	No	48 (19)	65 (5.1)	113 (7.4)	
Puncture site (mid calyx)	Yes	117 (46.2)	65 (5.1)	182 (11.9)	0.0005 **
	No	136 (53.8)	1213 (94.9)	1349 (88.1)	
Puncture site (upper calyx)	Yes	107 (42.3)	46 (3.6)	153 (10)	0.0005 **
	No	146 (57.7)	1232 (96.4)	1378 (90)	
Amplatz sheath size (Fr)	24	34 (13.4)	768 (60.1)	802 (52.4)	0.0005 **
	26	3 (1.2)	90 (7)	93 (6.1)	
	28	63 (24.9)	299 (23.4)	362 (23.6)	
	30	83 (32.8)	92 (7.2)	175 (11.4)	
	32	70 (27.7)	29 (2.3)	99 (6.5)	
Postoperative complications		253 (16.52)	1278 (83.47)	1531 (100)	
Modified Clavien-Dindo grades	I	62 (24.5)	0 (0)	62 (4)	0.0005 **
	II	107 (42.3)	0 (0)	107 (7)	
	3A	50 (19.8)	0 (0)	50 (3.3)	
	3B	22 (8.7)	0 (0)	22 (1.4)	
	4A	9 (3.6)	0 (0)	9 (0.6)	
	4B	2 (0.8)	0 (0)	2 (0.1)	
	5	1 (0.4)	0 (0)	1 (0.1)	
Stone-free status at 1 month	Yes	223 (88.1)	1190 (93.1)	1413 (92.3)	0.007 **
	No	30 (11.9)	88 (6.9)	118 (7.7)	

Previous history of PCNL {25 (9.9%) vs. 60 (4.7%)} and ureteroscopic lithotripsy (URSL) {59 (23.3%) vs. 89 (7%)} were significantly associated with complications, each with p-values < 0.01. The number of calyces involved, stone burden, presence of staghorn calculi, and the number of percutaneous accesses were also significantly different between the two groups and are summarized in Table 2.

**Table 3:** Postoperative complications classified according to the modified Clavien-Dindo grading system.

Grade of complications	No. of complications (N (%))
Grade 1	62 (4.04%)
Fever (> 38 ºC)	37 (2.41%)
Transient elevation of Serum Cr (> 0.5 mg/dl)	25 (1.63%)
Grade 2	107 (6.98%)
Blood transfusion	72 (7.70%)
Urinary tract infection requiring antibiotic	35 (2.28%)
Grade 3a	50 (3.26%)
Retention due to blood clots	19 (1.24%)
Renal hemorrhage requiring angioembolization	13 (0.84%)
Hydrothorax	8 (0.52%)
Pneumothorax	10 (0.65%)
Grade 3b	22 (1.43%)
Ureteric calculus	14 (0.91%)
Collecting system perforation	8 (0.52%)
Grade 4a	9 (0.58%)
Neighboring organ injury	1 (0.06%)
Acute renal failure	8 (0.52%)
Grade 4b	2 (0.13%)
Sepsis	2 (0.13%)
Grade 5	1 (0.06%)
Death	1 (0.06%)

In terms of surgical technique, the use of different puncture sites and larger Amplatz sheath sizes were significantly associated with complications. Higher Amplatz sheath size {24 Fr (13.4% vs. 60.1%), 28 Fr (24.9% vs. 23.4%), 30 Fr (32.8% vs. 7.2%), and 32 Fr (27.7% vs. 2.3%)} showed significant associations with complications (p = 0.0005).

Table 3 lists postoperative complications categorized according to the modified Clavien-Dindo grading system. Lastly, overall, 92.3% were stone-free. Postoperatively stone-free status at one month was significantly higher in patients without complications (93.1%) compared to those with complications (88.1%), with a p-value of 0.007.

The results of multivariate logistic regression analysis show that age >55 years {coefficient of 0.229 (p = 0.002)}, urinary tract infection (UTI) {coefficient of 5.330 (p = 0.0005)}, diabetes {coefficient of 8.091 (p = 0.0005)}, chronic kidney disease (CKD) {coefficient of 3.178 (p = 0.002)}, stone burden {coefficient of 0.001 (p = 0.002)}, percutaneous access number >1 {a coefficient of 11.464 (p = 0.002)}, Amplatz sheath size >30 Fr {coefficient of 0.408 (p = 0.015)} are a highly significant predictor of complications (Table 4).

**Table 4:** Multivariate analysis of predictors of complications. UTI: urinary tract infection; CKD: chronic kidney disease. * Significant at p < 0.05 but >0.01; ** Highly significant at p < 0.01; # No statistical significance at p > 0.05.

	B	SE	Wald	p-value
Age (>55 years)	0.229	0.074	9.608	0.002 **
Hematuria	1.844	1.028	3.218	0.073 #
UTI	5.330	1.486	12.856	0.0005 **
Diabetes	8.091	1.318	37.681	0.0005 **
CKD	3.178	1.006	9.979	0.002 **
Stone burden	0.001	0.000	9.336	0.002 **
No. of calyces involved	2.307	1.152	4.013	0.045 *
Percutaneous access number >1	11.464	3.760	9.298	0.002 **
Amplatz sheath (>30 Fr)	0.408	0.167	5.949	0.015 *
Puncture site (upper calyx)	-11.498	3.098	13.772	0.0005 **
Operative time	0.129	0.056	5.310	0.021 *
Constant	-70.968	3470.070	0.000	0.984

A greater number of calyces involved significantly increases the odds of complications. The odds ratio is 2.307 with a significant p-value (0.045), indicating a significant association. Longer operative time substantially increases the odds of complications (odds ratio is 0.129 with p = 0.021). The puncture site in the upper calyx is also a highly significant predictor, with a negative coefficient of -11.498 (p = 0.0005) suggesting that puncturing at the upper calyx decreases the log odds of the outcome by 11.498.

## Discussion

For individuals with a heavy burden of complicated renal stones, PCNL has become the preferred course of treatment. The safety of PCNL in diverse sufferer demographics, and several single-center investigations plus a multicenter global collective effort have shown its effectiveness and security in the healthcare environment [11-14]. It has been challenging to understand and compare data due to the lack of a defined reporting classification system. The updated Clavien-Dindo scoring system has been evaluated to resolve this problem and is now advised for use when describing endourological surgical results [15,16].

Our research sought to identify potential predictors of all potential PCNL problems. We found that several factors, including age, body mass index (BMI), comorbidities, staghorn calculi, number of percutaneous access punctures, stone burden, and operative time are associated with complications. Age >55 years, and multiple punctures were factors that substantially predicted a complication with a higher rate. Although it is possible that these elements are directly linked to a greater rate of complications, it is also possible that they serve as stand-ins for additional risk factors. The stone composition was not taken into consideration during the assessment, which is a limitation of this study. However, it is thought that postoperative problems may be more common with infected stones [17].

Previous studies have extensively investigated risk factors associated with postoperative complications following PCNL. Our findings are largely consistent with the existing literature, corroborating several known risk factors while also revealing novel insights.

Focused on the intraoperative and postoperative complications of PCNL, a study by Labate et al. observed that a higher number of comorbid conditions were associated with an increased risk of complications such as bleeding and infection [11]. Similarly, Michel et al. reported preoperative comorbidities, as assessed by the Charlson Comorbidity Index, were predictors of increased morbidity and mortality after PCNL [18]. Our study also identifies comorbid conditions such as diabetes and chronic kidney disease as significant risk factors for complications, reaffirming the role of baseline health status in surgical outcomes.

Falahatkar et al. and Sofikerim et al. retrospectively examined sufferers who had undergone open stone operations in the prior with those who had not, and they found no differences in postoperative problems [19,20]. In our study, since not enough patients had a prior history of open stone operations, we were unable to meaningfully examine the effect of prior open procedures on PCNL outcomes. However, previous history of PCNL and URSL were significantly associated with complications.

Kukreja et al. in their research found that larger kidney stones are associated with increased complications following PCNL [21]. Specifically, stones larger than 20 mm are linked to higher rates of bleeding and longer hospital stays. This finding was consistent with the results of our study, we noticed a higher post-operative bleeding rate in large burden (5000 mm3 - 20000 mm3 with>1 calyx involvement) stones. Larger and more complex stones, particularly staghorn calculi, were associated with increased operative times and higher complication rates. El-Nahas et al. noticed that lower pole stones are associated with less favorable outcomes, pose more challenges, and have higher complication rates post-PCNL [22]. In our analysis also complication rate was also higher in lower pole stones and patients with stones in multiple calyces.

A study by Yamaguchi et al. noted that significant blood loss during PCNL increased the risk of postoperative complications, including transfusion requirements and prolonged hospital stays [23]. A study by de la Rosette et al. investigated the impact of tract dilation methods on hemorrhage rates during PCNL and concluded that balloon dilation was associated with lower hemorrhage rates compared to telescopic and Amplatz dilation [24]. According to our observations, the size of the Amplatz sheath used for tract dilation had an impact on post-operative complications bleeding, larger size Amplatz sheath (30Fr) was associated with higher complication rates. Netto Jr et al. found that the number of access tracts significantly influenced complication rates, with multiple tracts increasing the risk of bleeding [25]. Similarly, we also found out that multiple percutaneous punctures and access tracts substantially predicted a higher complication rate with a higher Clavien-Dindo grade. Wu et al. in a meta-analysis suggested no significant difference in stone-free rates, complication rates, or blood loss between the supine and prone positions. So based on this meta-analysis, both positions may have similar complication rates [26]. In our study, since not enough patients had undergone supine PCNL, and patients were not randomized evenly in prone vs supine groups, we were unable to meaningfully examine the effect of both positions on PCNL outcomes.

Older patients are often associated with a higher prevalence of comorbidities and decreased physiological reserves, they may have compromised renal function, impaired wound healing, and decreased immune function, all of which can contribute to a higher risk of complications after PCNL. Higher BMI is often associated with increased surgical complexity due to factors such as difficulty in accessing the kidney, and longer operative times. Hence preoperative optimization of these factors is crucial. Higher complication rates in larger stone burdens may be attributed to longer operative times and multiple access tracts increasing the risk of bleeding. Whenever feasible, using smaller size sheaths may help to reduce the risk of hemorrhage. Minimizing the number of access tracts and utilizing alternative approaches, such as miniaturized instruments or flexible ureteroscopy, in cases of complex stone burden stones may help mitigate operative complications.

By examining complications and related risk variables, from proactively gathered databases from many centers, Labate et al reported a difficulty incidence of 20.5% [11]. They discovered that a higher mean Clavien-Dindo score was significantly predicted by the American Society of Anesthesiologists (ASA) physical status score. The Clavien-Dindo approach has the drawback of not taking long-term difficulties into account, which might affect subsequent care. Nonetheless, we concur with recent studies that the Clavien-Dindo system is a useful indication in the immediate postoperative period regarding the significance of disclosing complexity as a proxy for surgical quality [7]. Integrating the use of the updated Clavien-Dindo scoring system into clinical practice can facilitate communication among healthcare providers and improve patient care.

Limitations of the study

While our study contributes valuable insights into the risk factors for postoperative complications of PCNL, it is essential to acknowledge its limitations. Retrospective analysis is the major limitation of our study. The usage of mathematical formulas to quantify renal function instead of nuclear renography and glomerular filtration rate (GFR) estimates also has its own inherent bias. A significant number of patients for whom the data were missing or incomplete might have significantly influenced the final outcomes as well. The stone composition was not analyzed. Infected stones might have added to more complications as urine culture may not be sufficient enough to document infections harboring within the stone. The follow-up period was too short. A longer follow-up period could throw more light on long-term complications as well. Multiple surgeons, though experienced enough in both supine and prone PCNLs, could be a confounding factor with regard to complications. A larger multicentric prospective study would be much more representative, especially in diverse demographic renal stone patients.

Future research should aim at prospective studies with larger sample sizes and multi-center collaborations. Furthermore, investigating the role of emerging technologies such as robotic-assisted PCNL and advanced imaging modalities may offer additional insights into optimizing patient outcomes in PCNL procedures.

## Conclusions

Standard PCNL has a low overall complication rate and is still an excellent modality of treatment with mini-PCNL/RIRS emerging as the preferred option for large-burden kidney stones. The likelihood of a serious complication is greatly enhanced by old age, associated comorbidities (diabetes and chronic kidney disease), multiple percutaneous access tracts, and the use of large-size Amplatz sheaths. Preoperative optimization, reduction in percutaneous access tracts with smaller Amplatz sheath (24 Fr), and shorter operative time will help to reduce the complications rate.
